# A Low-Cost Tracking System for Running Race Applications Based on Bluetooth Low Energy Technology

**DOI:** 10.3390/s18030922

**Published:** 2018-03-20

**Authors:** David Perez-Diaz-de-Cerio, Ángela Hernández-Solana, Antonio Valdovinos, Jose Luis Valenzuela

**Affiliations:** 1 Signal Theory and Communications Department, Universitat Politècnica de Catalunya, Esteve Terrades 7, 08860 Castelldefels, Spain; dperez@tsc.upc.edu (D.P.-D.-d.-C.); valens@tsc.upc.edu (J.L.V.); 2 Aragon Institute for Engineering Research (I3A), University of Zaragoza, 50018 Zaragoza, Spain; toni@unizar.es

**Keywords:** BLE, RFID, running races, tracking, monitoring, Bluetooth 5

## Abstract

Timing points used in running races and other competition events are generally based on radio-frequency identification (RFID) technology. Athletes’ times are calculated via passive RFID tags and reader kits. Specifically, the reader infrastructure needed is complex and requires the deployment of a mat or ramps which hide the receiver antennae under them. Moreover, with the employed tags, it is not possible to transmit additional and dynamic information such as pulse or oximetry monitoring, alarms, etc. In this paper we present a system based on two low complex schemes allowed in Bluetooth Low Energy (BLE): the non-connectable undirected advertisement process and a modified version of scannable undirected advertisement process using the new capabilities present in Bluetooth 5. After fully describing the system architecture, which allows full real-time position monitoring of the runners using mobile phones on the organizer side and BLE sensors on the participants’ side, we derive the mobility patterns of runners and capacity requirements, which are determinant for evaluating the performance of the proposed system. They have been obtained from the analysis of the real data measured in the last Barcelona Marathon. By means of simulations, we demonstrate that, even under disadvantageous conditions (50% error ratio), both schemes perform reliably and are able to detect the 100% of the participants in all the cases. The cell coverage of the system needs to be adjusted when non-connectable process is considered. Nevertheless, through simulation and experimental, we show that the proposed scheme based on the new events available in Bluetooth 5 is clearly the best implementation alternative for all the cases, no matter the coverage area and the runner speed. The proposal widely exceeds the detection requirements of the real scenario, surpassing the measured peaks of 20 sensors per second incoming in the coverage area, moving at speeds that range from 1.5 m/s to 6.25 m/s. The designed real test-bed shows that the scheme is able to detect 72 sensors below 600 ms, fulfilling comfortably the requirements determined for the intended application. The main disadvantage of this system would be that the sensors are active, but we have proved that its consumption can be so low (9.5 µA) that, with a typical button cell, the sensor battery life would be over 10,000 h of use.

## 1. Introduction

According to the statistics analyzed by Running USA, a nonprofit group that provides industry analysis to help promote long-distance races, during 2016 more than 30 thousand races were held along the U.S. with near 17 million of runners finishing them [1]. In fact, nowadays, the success is such that someone based in, or near a big city, has a lot of chances to be spoilt for choices when it comes to race options (marathons, half marathons, 5 Ks, obstacle races,…). This exponential increase rises new related technological problems, for example, how to track and manage the great amount of runners in a race.

Traditionally, the tracking systems used in this kind of events are based on RFID. These systems have been present in thousands of events since the Berlin Marathon in September 1994 [2] and are the tracking systems used in Spain as well as in other countries. These systems require each runner to wear a passive RFID sensor (tag). Additionally, the organizers must place the corresponding readers at the points where is necessary to control the time. Generally, the antennae of these readers are placed under anti-slip mats or ramps over which all the participants must go through. The tags, on their side, are usually placed on the participants’ shoes to allow a minimum distance between the tag and the reader antenna. The advantages of these systems is that are well-proven, high precision and accurate location solutions [3,4]. The sensors are low-cost and passive so they do not need any type of maintenance. The participants can rent the sensors before each race or buy them in property and use them for an uncountable number of races. However, the main disadvantages are present on the organizer side. These solutions are not suitable for small competitions and are expensive to buy for small races [5]. Additionally, they are bound to specific hardware and all the components of the system must belong to the same ecosystem in order to work. Third-party or mixed solutions are hard to configure, and it is even common that a person from the tracking equipment company comes to monitor the deployment and handle the tracking system, increasing in this way the cost. Additionally, the deployment is not very flexible: a single 205 × 100 × 1 cm mat weighs around 12 kg and this does not take into account the equipment that must be connected to it. Usually more than one mat is needed per checkpoint and, moreover, placing these checkpoints can be difficult depending of the nature of the race, for example, in cross-country races. Some low-cost RFID-based platforms have been proposed for jogging and running events. In order to reduce the cost, these systems use low-frequency (LF) RFID devices and a low-cost, but whereas typical race systems use ultra-high frequency (UHF) RFID readers, in which the range is 5–30 m, LF RFID readers present a range of only 10–20 cm [6]. Other solutions propose an identification mechanism that uses Ultra Wide Band (UWB) ranging information to improve the speed of identification [7], but increasing the complexity and the cost of the system. In any case, RFID technology, as most of radiofrequency technologies, does not intrinsically provide aptitudes for high spatial resolution and to identify the time of presence of the tag on a specific place with high precision, particularly in complex propagation environments. In fact, the RFID signal does not provide intrinsically a better spatial resolution than the Bluetooth signal. In order to get good spatial accuracy in both technologies, it is necessary to use complex systems, with several directional antenna or antenna arrays, obtaining the range information from parameters not always easily obtained, as the received signal strength indication (RSSI), the time-difference-of-arrival (TDOA), the phase-difference-of-arrival (PDOA) or the round trip-of-flight (TOF), and determining the tag location by utilizing multiple known reference points using methods as multilateration, multiangulation, etc. [8]. In typical systems developed for racing applications, the spatial accuracy is provided in RFID systems by casting the send and receive antennas in thin tartan mats, which are located at the finish line and other timing locations. Since passive tags are used, the reader only receives the ID-number sent by the tag when they are very close each other (when the runner is over the mat). If a higher accuracy is required, a more sophisticated technology has to be used, for instance, photofinish systems. This kind of systems are too expensive to be considered for local jogging and running events, in which there is little budget. There are also previous experiences in which Bluetooth Low Energy (BLE) has been used to provide crowd mobility detection by using a smartphone, with detectability rates of up to 90% [9]. Additionally, BLE has been proved to provide a good tradeoff between discovery latency and energy consumption [10,11].

In this paper we propose a BLE-based tracking system which can be used as an enhancement or even as an alternative to the RFID-based solutions in scenarios in which it is not necessary to obtain precise time measurements, such as the presence control of the runners through intermediate checkpoints. Spatial resolution can be improved by reducing the coverage distance, which can be done in RFID by using passive tags, but also in Bluetooth by adjusting the power transmitted by the advertisers. In any case, if the range of coverage is decreased, there is less time to discover all the advertisers (runners) when they pass through the checkpoint. It is therefore necessary to achieve a balance between both aspects. Our proposal seeks to obtain a simple, easy to handle and cheap system that allows detecting reliably the passage of the runners by a certain point with a precision in the measurement of time that is not critical, and can be of the order of a few seconds. Whereas RFID readers are specific and expensive devices, particularly passive RFID readers, in our proposal the Bluetooth reader is a simple Android smartphone, in which is easy to develop an application to configure and manage the system and provides accurate enough position information thanks to its integrated GPS receiver and a communication link with the server via a 3G/4G cellular network. Furthermore, Bluetooth offers the possibility of transmitting additional information to the mere identification of the runner, such as biometric data.

First, we will present the system architecture followed by the basics behind the BLE mechanisms which enable the communications of all the system sensors. Specifically, we consider two low complexity schemes based on the advertisement processes allowed in BLE. The first one uses the non-connectable undirected advertisement mode. The second is a modified version of scannable undirected advertisement mode. This option is based on the ability to stop the transmissions using one of the new Bluetooth 5 capabilities that was previously analyzed by the authors [12]. We also consider that real devices present non-idealities in the scanning process that reduce the discovery capabilities [12,13]. After the system description, we will analyze the requirements of this type of events using real data obtained from the 2017 Barcelona Marathon. With this analysis we will obtain real mobility patterns of the runners such as speed or runner density at different points of the race. These values will be determining to set the requirements of the system under real conditions. Then, by means of simulations, we will check if the proposed system is able to meet the scenario requirements. We will determine the viability of detecting all the runners and the more appropriate parameters to be configured, such as coverage range, time interval between transmitted packets, etc. In order to increase the capacity of the system, the use of several readers is also considered. Although, in the present discussion the final application is the tracking of runners during a race, the results can easily be extrapolated to other applications such as other kinds of races, tracking kids, cattle control, drone tracking and flight assistance, product chain control in an Industry 4.0 factory, etc. 

The proposed alternative offers also additional capabilities not present in the current RFID solutions as can be the real time monitoring of the participants’ health constants. Several works have studied the not so rare cases of deaths during sports events. Their results show that 1.74 per 100,000 athletes have suffered from a sudden cardiac death in triathlons [14] and this number can even be increased to 1.9 per 100,000 runners in marathons [15], so, in this way some serious illnesses and even deaths could be prevented with the use of the proposed system.

## 2. System Architecture

**Figure 1:**
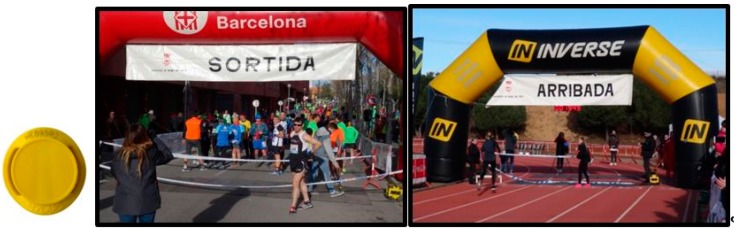
RFID Solution.

As explained before, the systems based on RFID require that each runner buy or rent his/her own RFID tag (see Figure 1). Additionally, the organizers must install ramps or mats at the main race points, like the ones depicted on the right of Figure 1 to control when each participant passes through these points.

**Figure 2:**
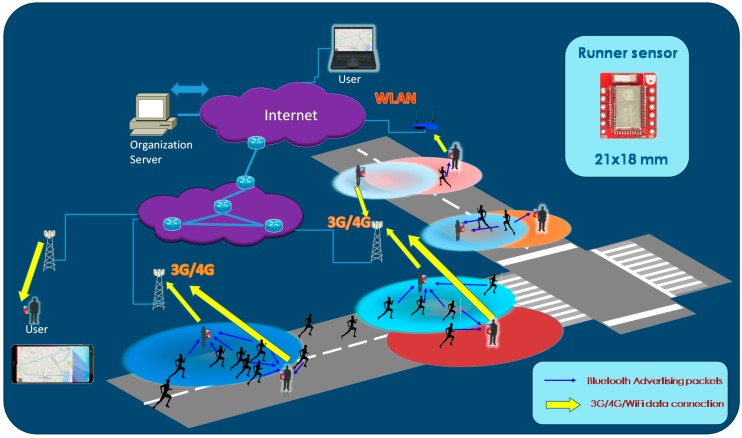
System deployment.

In our proposal, depicted in Figure 2, the participants are also required to wear a sensor, in this case, based on BLE. The dimensions of the sensor are similar to the RFID equivalent. However, the RFID ramps or mats are substituted by an application on a smartphone. The organizers usually designate several people to stay along the track for security reasons and to control the successful development of the race. Each, or some of this part of the staff members, as many as necessary, should wear a smartphone with the tracking application. These smartphones will act as receivers, known as scanners in BLE terminology.

The devices worn by the participants, on their side, transmit periodically a BLE beacon (advertisement) with the participant ID and any other supplementary data (blood pressure, heart rate, etc.). When they enter into the coverage area of one of the controlling member scanners these packets will be eventually received.

In the most basic mode, using the BLE *non-connectable undirected advertisement* mode it is necessary to transmit the frames frequently enough to ensure that the sensor it is detected. After a successful reception, the application will append to this information the current location of the smartphone and timestamp obtained by GPS/GLONASS/Galileo and it will be stored on the device and sent in real-time using an available data connection (3G/4G/WLAN) to an organization server on the cloud.

On an enhanced mode, we propose to use the *BLE scannable undirected advertisement mode*. In this case, after a successful reception of an advertisement, the scanners shall answer with another short packet which is used to acknowledge the reception. Thus, using one of the new Bluetooth 5 capabilities, once the advertiser receives this acknowledgement we force it to enter into a stand-by mode for a while to reduce interferences and, in this way, increase the capacity of the system as we will demonstrate later. Also, in order to enhance the performance of the system, several scanners are allowed to cover the same area as depicted in Figure 2.

The organizers may eventually allow the access to these data to authorized people, for example, friends or family of the participants so they can know during the progress of the race their precise location and race position in real-time. Additionally, based on the monitored data received it is also possible to generate warnings if anomalous conditions of a determined participant are detected. Thus, the security staff could intervene to avoid dangerous situations, like cardiac arrests or sudden deaths.

## 3. BLE Fundamentals Review

In 2010, the Bluetooth Special Interest Group (SIG) incorporated the Low Energy specification into the Bluetooth specifications. Since then, several enhancements and modifications through various revisions have been performed until the last one, Bluetooth 5, which was released in December 2016. Bluetooth devices operate in the 2.4 GHz Industrial Scientific Medical (ISM) unlicensed band. Although classic Bluetooth allows higher data rates, BLE compliant devices allow wireless communications between enabled devices with up to 1 Mbps over the air data rates (2 Mbps on Bluetooth 5).

In this section we review the basics of BLE technology, especially those aspects related to the intended application, particularly, the device discovery procedures. For further details about BLE, a good summary can be found in the work by Gomez et al. [16], and the publicly available specifications [17].

**Figure 3:**
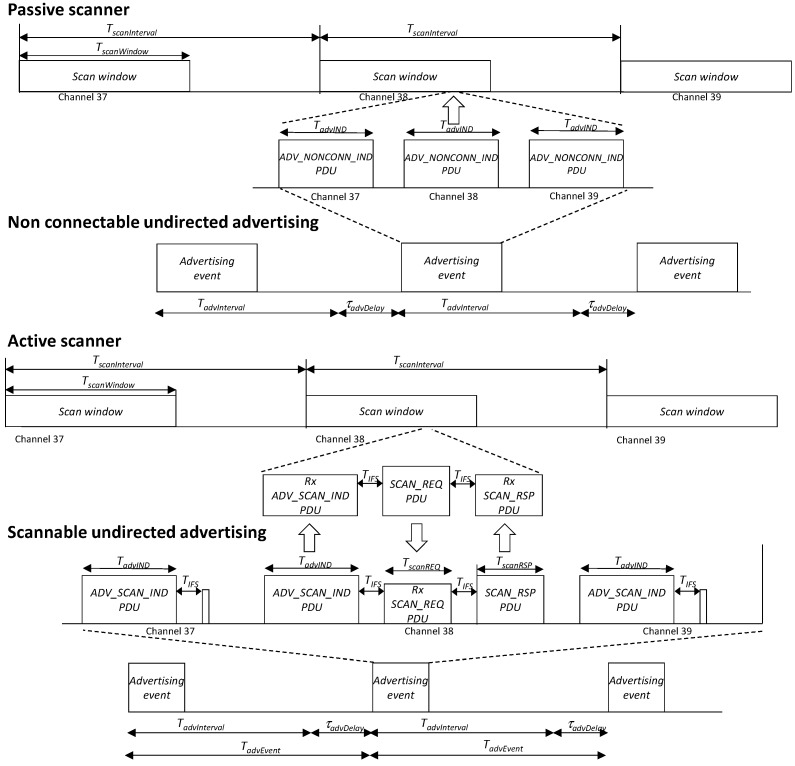
Non-connectable and scannable undirected advertisement detection procedure.

The basic communication mechanism to transmit data and discover devices in BLE is based on broadcasting messages periodically (advertisement events, see Figure 3). These frames are transmitted, by an advertiser, on three predetermined channels: 37, 38 and 39, although a mask can be applied to select any combination of these three channels. The advertisement events are transmitted every T_advEvent_, a period which is composed by a fixed part, known as advertisement interval (T_advInterval_), and a random time between 0 and 10 ms, T_advDelay_. This randomness is necessary to minimize consecutive collisions between several advertisers. The amount of user data sent inside an advertisement can be comprised between 1 byte and 26 bytes. So, the duration of the frames (T_advIND_) must be in the range from 176 µs to 376 µs. This capacity is more than enough to transmit the monitoring data (blood pressure, heart rate, etc.) that we have commented previously.

On the receiver side, the scanner listens on one of the three advertisement channels during a configurable time period called scan window (T_scanWindow_). After a scan interval (T_scanInterval_), a time period at least as long as T_scanWindow_, the scanner switches to the next advertisement channel in a round-robin fashion. Real devices are not able to switch between frequencies instantaneously and introduce a blind-time during while any received frame is not decoded. More information about this effect and others present in real devices can be found on [12,13].

The standard specifies several advertisement modes, in this work we will consider only two of them: *non-connectable undirected advertisements* and *scannable undirected advertisements*. When applying the first one, the advertiser just broadcasts the advertisements periodically on one direction without waiting for a response. With *scannable undirected advertisements* after the end of the transmission of an advertisement frame, the advertiser waits for 150 µs (T_IFS_) for a scan request. The duration of this frame (T_scanREQ_) is 176 µs. This request shall be transmitted on the same frequency by a scanner which has received the last transmitted advertisement. To achieve this, the scanner shall be configured to be in active mode. Once a scan request is successfully received, the advertiser broadcasts, after a T_IFS_, another frame (scan response) on the same channel. The duration of this frame (T_scanRSP_) is configurable between 128 µs (no data) and 376 µs. It is important to remark that the scan request is addressed specifically to the advertiser that triggered the procedure.

Obviously, when using the *scannable undirected advertisement* mode, the amount of frames over the air increases, and so do the probability of collisions and interferences between the system devices. However, with Bluetooth 5 a new event, not available in previous versions, is generated and passed to the upper layers when the advertiser receives a scan request. In our proposal, we will use this event to put the advertiser into a stand-by mode and stop any further transmissions until a configurable timeout has passed. After this timeout, the device is reactivated to be detected again by the scanner on the next checkpoint. Thus, the capacity of the system could be increased. The advertisers automatically send a scan response after a successful scan request. To reduce the amount of interference, we configure the devices to send an empty scan response (T_scanRSP_ = 128 µs).

Another point to be considered when using *scannable undirected advertisements* is the possibility that two scanners simultaneously transmit a scan request. The specifications suggest an example of a backoff algorithm for these special cases, however is the manufacturer which decides with algorithm to implement. Due to this degree of freedom, in our application, when we propose to use more than one scanner to improve the capacity of the system under unfavorable BLER conditions, we will force the scanners to be unsynchronized. In this way, each scanner should be listening for advertisements in a different frequency than the others and the backoff algorithm should not be triggered.

## 4. Application Requirements

**Figure 4:**
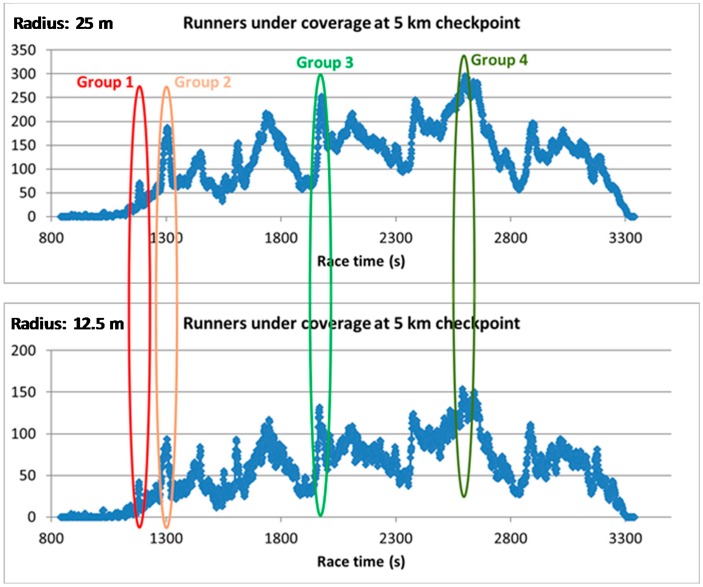
Runners under coverage at 5 km checkpoint for different coverage radii.

To obtain the application requirements, we have analyzed the data from the 2017 Barcelona Marathon [18]. The Barcelona Marathon is a popular race, with near 15,000 participants in the last edition. The stored data not only provides the race and real finish times, but also the participants’ time at different checkpoints of the race. Without loss of generality, we will obtain the runners patterns and different metrics analyzing the 5 km checkpoint data, see Figure 4.

The first metric we can extract from the data is the speed of the athletes, it ranges from 1.51 m/s up to 6.25 m/s. This value will allow us to calculate easily the amount of time under coverage of the sensors for a determined cell radius and also to work out the suitable off-periods of the sensors when they are detected to reduce interferences. Thus, the timeout before reactivating an advertiser can be fully configurable. Given the separation between control points, measurements can be useful to set a secure value for the maximum time that a device can remain without sending advertisers. On the other hand, an estimation of the holding time under the coverage area could be useful to set the minimum value for this time in order to prevent unnecessary interferences. For example, an athlete running at 6.25 m/s, when entering into a 25 m diameter coverage cell, could turn off the transmitter once detected for 4 s without interfering with other devices, whereas a slower runner (1.5 m/s) requires 17 s to be out of the coverage area. A minimum threshold of 30 s can be a suitable option.

The second important metric is related to the system capacity, i.e., the number of sensors the scanner should detect under its coverage. Obviously, if the cell radius is increased, the number of participants under coverage is also increased. In Figure 4, we have highlighted four runner groups, which are some of the most interesting points of the figure. With the values selected in this case: group 4 and a cell radius of 25 m, which is the worst scenario, the number of sensors to be detected would be near 300. However, it is necessary to take into account also the speed of each group, because this will determine the time under coverage and the successful detection probability.

**Figure 5:**
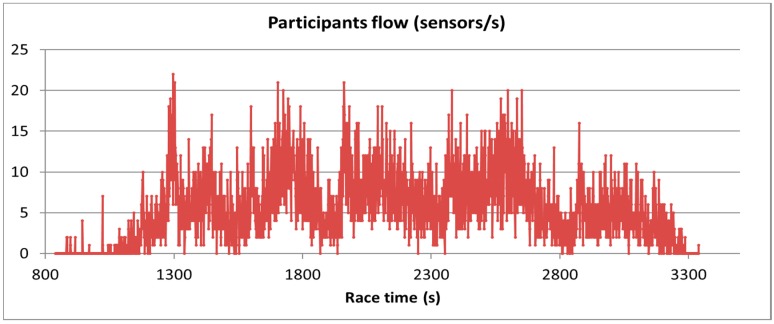
Sensor flow at the 5 km checkpoint.

When using the Bluetooth 5 new event notification to stop the advertisers once they have been detected, the previous metric loses its meaning and the flow of participants (sensors entering per second into coverage) should be used instead. Figure 5 represents this metric at 5 km checkpoint. As can be seen, the maximum number of sensors to be detected simultaneously is around 20 sensors per second.

## 5. Tests and Results

Once determined the mobility patterns of the participants, in this section we evaluate if the proposed sensor network is suitable in terms of coverage, energy consumption, capacity, detection probability, etc.

### 5.1. Coverage Test

**Figure 6:**
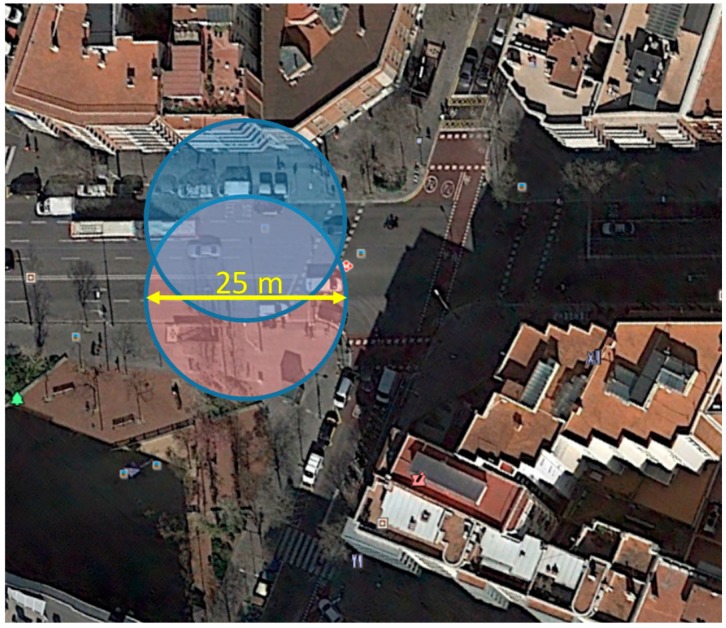
Cell coverage.

The coverage area of the different cells (see Figure 6) can be adjusted changing the transmitted power of the sensors. In this way, we can control the precision of the tracking system and the number of sensors which could be present in each cell.

We have measured on the field the coverage for a static advertiser and a moving one at 25 km/h for all the range of transmitted power allowed by the device under test. The results are shown on Table 1 and go from coverage areas of 2 m up to 400 m.

### 5.2. Current Consumption Results

RFID tags are usually passive and require very low maintenance. BLE sensors, on the other hand, are usually battery operated, so, to reduce their maintenance, the power consumption of the devices should be kept to a minimum to be an acceptable alternative over RFID. BLE consumption when the device is not transmitting is so low that it can be neglected, so the main energy consumption depends on the number of transmissions done and their duration, i.e., the advertising interval and the packet size. We have tested the consumption of the devices at the laboratory taking into account these parameters and the results obtained are shown in Table 2. For example, with a CR-2032 lithium battery which has a 210 mAh capacity, the sensor battery life would be over 10,000 h of use.

### 5.3. Flow Absorption Test

**Figure 7:**
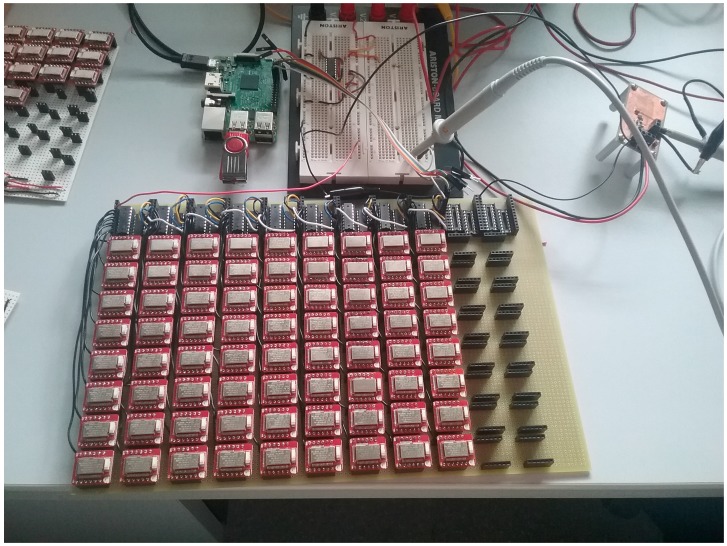
Test-bed setup.

We also designed a laboratory test to determine if the system was able to detect and stop enough transmitters to meet the requirements presented on Section 4. Our proposal suggests to turn off the advertisers when receiving a scan request using the new Bluetooth 5 notification event. However, at the moment of doing the experiment, we did not have real Bluetooth 5 devices to realize the test. So, we implemented the set up shown in Figure 7 which emulates a more restrictive case. Thus, if the results obtained fulfill the requirements so will the original proposal.

This test-bed is composed of 72 Redbear nano [19] BLE advertisers. The scanner is a Raspberry pi 3. We can control the power lines of the advertisers through the Raspberry pi GPIO pins which are connected to several shift registers in a daisy chain configuration. Each shift register controls a row of eight advertisers. In this case, the advertisers cannot disconnect themselves, so we map the position and address of each of the advertisers and is the Raspberry pi which disconnects them. When the Raspberry-integrated Bluetooth transceiver receives an advertisement, it sends the corresponding scan request. Then, if the scan response is received successfully, it turns off the advertiser involved in that frame exchange.

**Figure 8:**
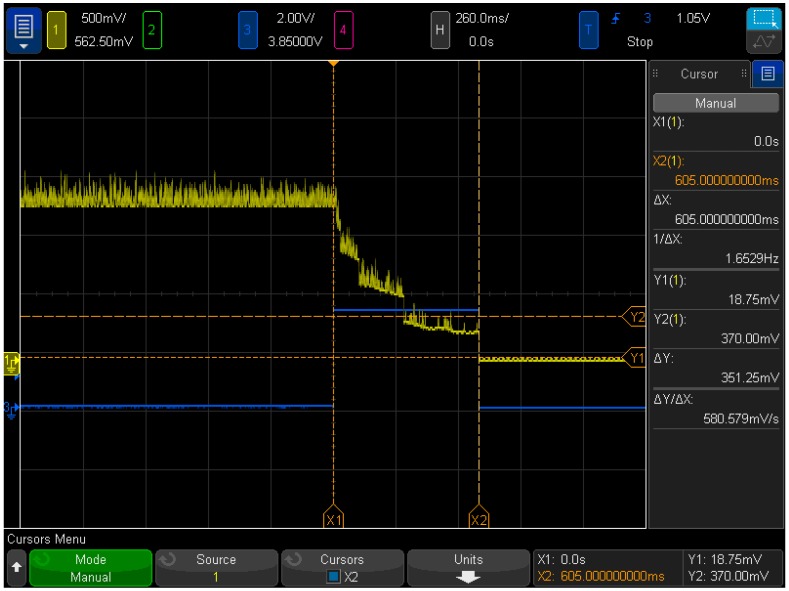
Detection and disconnection of 72 advertisers.

To measure the response of the system we monitored the consumption of the whole board with a current monitor connected to an oscilloscope. An example of the results for this experiment is shown in Figure 8. There it can be seen that the system is able to disconnect the 72 devices in around 600 ms. To be suitable for the runner tracking application, it was necessary to stop up to 20 sensors per second, so although the emulated scenario is more restrictive it fulfills the conditions more than enough for the requirements.

### 5.4. Simulation Results

In this section we will validate by means of simulations if the system is able to accomplish all the requirements presented in Section 4. The simulator is programmed in C++ and takes into account all the Bluetooth parameters described in Section 3. Performance statistics are obtained by averaging up to 10,000 coverage time intervals. In all the cases studied, we have considered that the scanner is scanning continuously (T_scanWindow_ = T_scanInterval_ = 500 ms), but taking also into account the limitations of real devices [12,13]. Following the notation described in those studies, the scanners considered in these simulations follow the behavior of type 2 devices. Additionally, other variables related to each scenario are also considered, i.e., the Block Error Rate (BLER) applied to each frame involved in the simulation, the number of sensors/advertisers (N_dev_), the cell radio (R), the time under coverage of the sensors (T_cov_) or the number of scanners present.

The viability of the application has been evaluated for each of the four athlete groups highlighted in Figure 4. Each group runs at a different speed in decreasing order: 4.16 m/s, 3.84 m/s, 2.5 m/s and 1.92 m/s, therefore, for a fixed coverage radio (R), the time (T_cov_) and number of devices (N_dev_) under coverage depend on this speed. We have considered two possible values of R suitable for the intended application: 12.5 m and 25 m. N_dev_ for each group can be extracted from Figure 4 and T_cov_ can be calculated for each R using the group speed.

**Figure 9:**
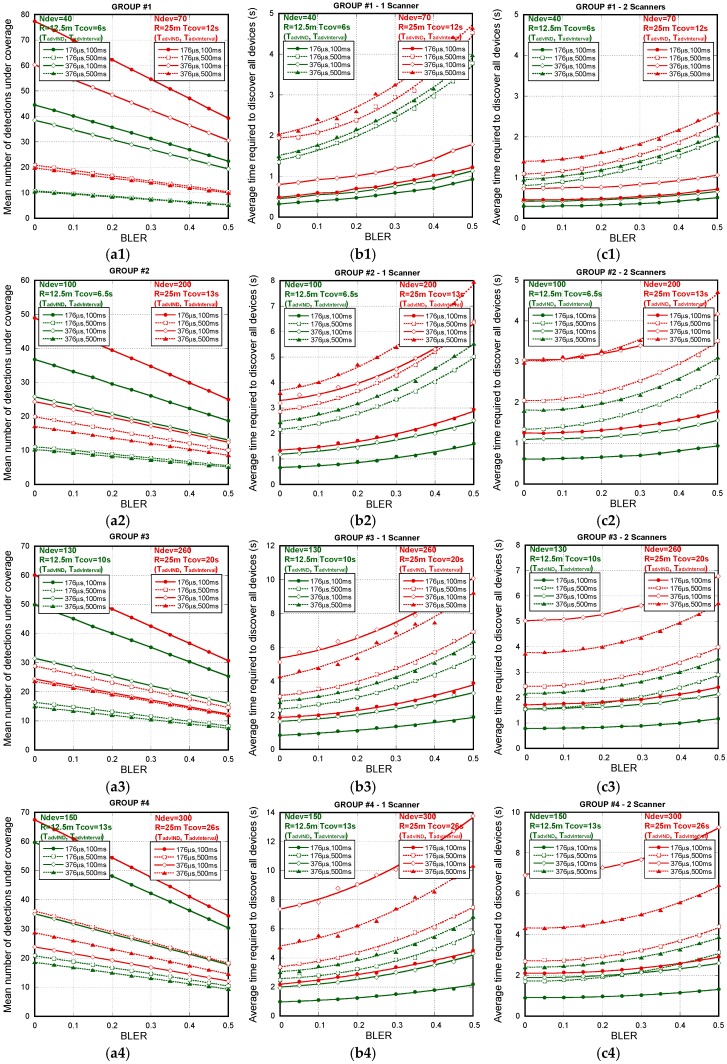
*Non-connectable undirected advertisement scheme*. Rows ((**1**) to (**4**)) represent results for each of the four athlete groups running at different speeds. First column (**a**) represents the mean number of detections under coverage under a single scanner coverage. The second and third columns show the average time required to discover all devices under a single scanner coverage (**b**) and under two scanners (**c**) overlapped coverage, respectively, for several values of BLER, T_advIND_ and T_advInterval_.

In the first test batch, which corresponds to the results shown in Figure 9, the system under analysis uses the *non-connectable undirected advertisement*. In this case, the advertisers are not stopped when they are detected, so when they pass through the coverage area (T_cov_) of a scanner they can be detected several times. 

**Figure 10:**
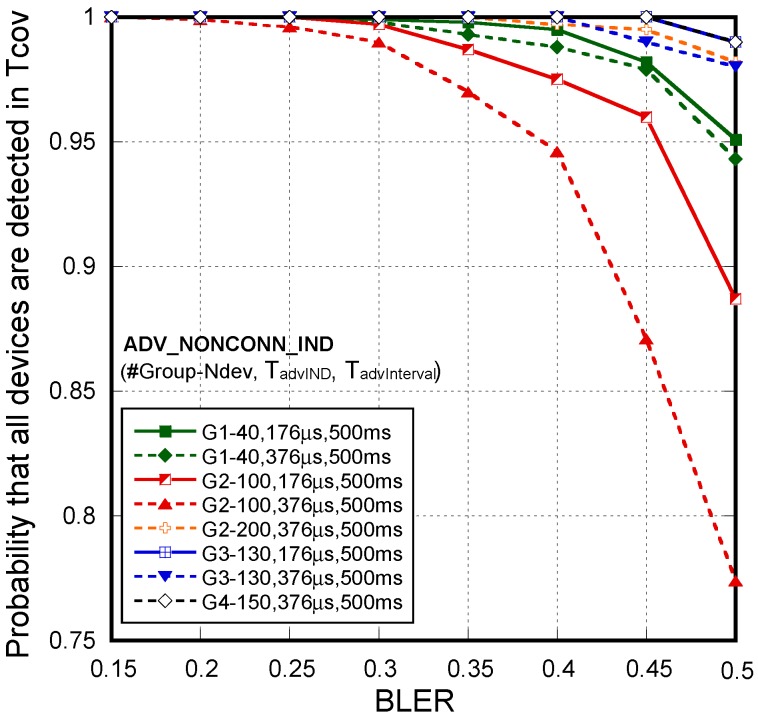
*Non-connectable undirected advertisement scheme*. Probability that all devices are detected under a single scanner coverage. Comparison between Group #1 (**1**), #2 (**2**), #3 (**3**) and #4 (**4**). Cases without a 100% of detection probability.

Besides, as there are not scan request/response interchanges, when considering several scanners, they may work freely, i.e., it is not necessary to force them to be scanning on different frequencies. That is, no coordination between scanners is required. Rows in Figure 9a–c represent the results for each of the four athlete groups highlighted in Figure 4. The first column of Figure 9 (a1–a4) shows this fact, depicting the mean number of detections of the same device for different values of BLER, packet sizes/duration (T_advIND_) and advertising interval (T_advInterval_) under a single scanner coverage. There we can see that a single advertiser can be detected from 5 to 75 times depending on the selected parameters. The second (b1–b4) and third (c1–c4) columns represent the average time required to discover all the devices within the coverage area considering one and two scanners, respectively, for the same parameters variations. Starting by the one scanner configuration, as per the results, the time to detect all the devices under coverage for every case is always below T_cov_. The lower coverage radio (R = 12.5 m) offers lower discovery times thanks to a minor number of devices competing for detection. The best results are obtained always for the smaller advertising intervals (T_advInterval_ = 100 ms) and, no matter the advertising intervals, smaller frame sizes (T_advIND_ = 176 µs) offer the best results. Nevertheless, in order to assess the viability of the proposal or to choose the best configuration parameters of the system, not only the average time required to discover all the devices is relevant, but also its distribution. For this reason, connected with results of Figure 9, Figure 10 shows the probability that all the devices are detected when only one scanner is used. Figure 10 only represents the cases without a 100% of detection probability. When R = 12.5 m is considered, not all devices can be detected when the error rate exceeds the 20% threshold and higher values for advertising intervals (T_advInterval_ = 500 ms) are configured. For such long advertisement intervals, channel errors and collisions make very difficult the successful reception of the frames in short T_cov_. It is important to remark that when R = 25 m is considered, even in very pessimistic conditions, e.g., 50% of all transmitted frames are received erroneously, the system is able to detect all the sensors under T_cov_, no matter the speed of the group, frame size or advertising interval employed. However, on the contrary to the conclusions obtained for R = 12.5 m, another fact to highlight is that the duration of the frames gets especially relevance for high sensor densities. The mean delay for T_advIND_ = 376 µs is greater for shorter advertising intervals than for longer ones. In these scenarios the number of collisions is considerably high, so increasing the advertising interval can help to reduce the time to detect all the devices present. This effect can be observed, for example, looking at the last subfigure of Figure 9(b4) and noticing how the red lines are switched. In those cases, it is better to increase the advertising interval.

The third column of Figure 9 shows that the introduction of just one more scanner can reduce significantly the average time needed to detect all the devices under coverage for every case. On the other hand, the system is able to detect all the sensors under T_cov_ no matter the speed of the group, frame size or advertising interval employed, with the exception of the N_dev_ = 100, T_advIND_ = 376 µs and T_advInterval_ = 500 ms, with above 99%.

To better illustrate the system performance versus parameter settings by using the *non-connectable undirected advertisement* function of the number of sensors/advertisers (N_dev_), Table 3 compares the required T_cov_ for detecting all the sensors (discretized in steps of one second), and the average time required to discover all the devices considering one and two scanners, for BLER = 30%. Note that BLER = 30% is a very pessimistic or conservative scenario. The probability that a device is affected by a continuous BLER of 30% along all the scanner coverage, in all the scanner frequencies and with the two scanner devices, is very low. Even in this context, the system performance is really good for T_advIND_ = 176 ms and T_advInterval_ = 100 ms, no matter one or two scanners are configured. For long advertisement intervals and frame sizes, two scanners clearly outperform results obtained with only one scanner.

The second tests batch corresponds to the results for *interrupted scannable undirected advertisements* using one and two scanners respectively and stopping the advertisers once they receive a scan request. Results are also obtained for different values of BLER, packet sizes/duration (T_advIND_) and advertising interval (T_advInterval_) under a single scanner and two scanners coverage. It is necessary to recall that in this mode we force the receivers to be scanning always in different frequencies in order to avoid collisions between the scan requests from the two scanners. In high-density networks, the backoff algorithm can also be unnecessarily activated when the scan request and scan response frames are not received due to BLER or collisions with advertising frames from other devices, increasing detection delay. Thus, as suggested in a previous work [3], backoff is deactivated in this application. In all the cases, scan request and scan response frames are set to the minimum values in order to minimize collisions. That is, T_scanREQ_ = 176 ms, T_scanRSP_ = 128 ms.

**Figure 11:**
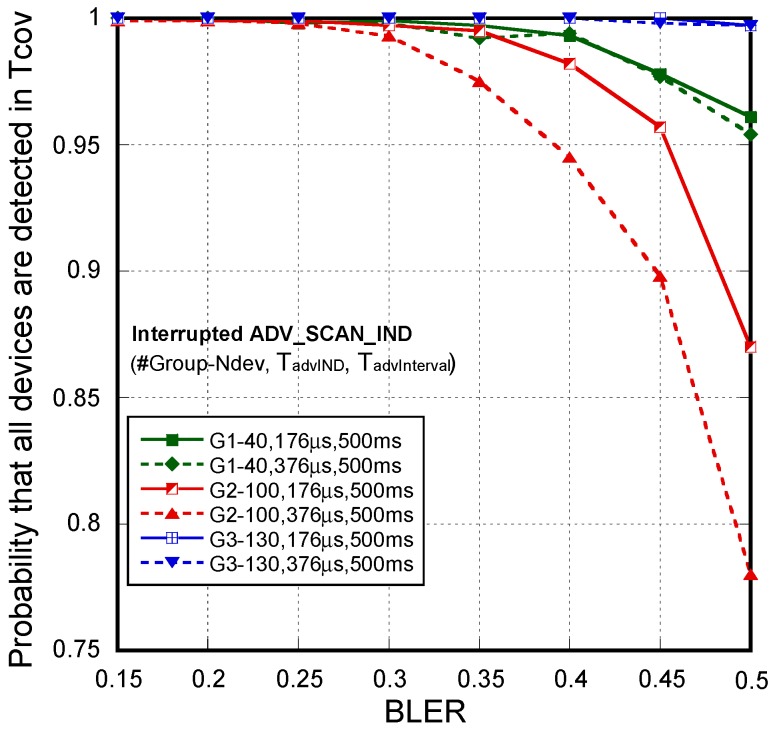
*Interrupted scannable undirected advertisement scheme*. Probability that all devices are detected under a single scanner coverage. Comparison between Group #1 (**1**), #2 (**2**), #3 (**3**) and #4 (**4**). Cases without a 100% of detection probability.

First, as with *non-connectable undirected advertisement*, the performance of the system has been evaluated for each of the four athlete groups highlighted in Figure 4. In this case, taking in consideration an unrealistic but high demanding assumption, the number of devices N_dev_ are assumed to arrive simultaneously in the coverage area. Under this assumption, Figure 11 shows the probability that all the devices are detected when only one scanner is used. Figure 11 only represents the cases without a 100% of detection probability. The probability when two scanners are considered is 100% in all the cases. By comparing Figure 10 and Figure 11, we can obtain similar conclusions about system parameter settings. For longer advertisement intervals, channel errors and collisions make very difficult the successful reception of the frames in short T_cov_. A lower coverage radio (R = 12.5 m), even though a lower number of devices is under coverage, may not guarantee the application requirements.

As in Table 3, Table 4 allows us to better illustrate the system performance versus parameter settings by using the *interrupted scannable undirected advertisements*. Results are obtained in function of the number of sensors/advertisers (N_dev_), considering one and two scanners and for BLER = 30%. Firstly, we can see that the proposed scheme outperforms *non-connectable undirected advertisement* in all the cases. Even under this high demanding assumption (the N_dev_ devices enter in the coverage area simultaneously), the system performance is good for shorter advertisement intervals, no matter whether one or two scanners are configured. For longer advertisement intervals and frame sizes, two scanners clearly outperform the results obtained with only one scanner. 

Nevertheless, this comparison is certainly unfair for the *interrupted scannable undirected advertisement*. When this scheme is used, the advertisers are stopped once they receive a scan request, although they complete the event by transmitting the scan response. Looking at Figure 5, we notice that the maximum number of sensors that simultaneously enter the coverage area is up to 20 sensors per second. Thus, if sensors are detected and turned off in less than a second, the number of competing sensors is always limited to 20 in all the groups, no matter the coverage area.

To better analyze the proposed scheme, Table 5 and Table 6 show the T_cov_ required for detecting all the sensors (receive the advertisement frame by one of the scanners) and the T_cov_ required for stopping all the sensors (receive the scan request frame by the advertiser) for N_dev_ = 20, 40 and 70, different values of BLER, packet sizes/duration (T_advIND_) and advertising interval (T_advInterval_), considering one and two scanners, respectively. Both values are integers because we have considered a granularity of one second. T_cov_ required for stopping all the sensors can be equal or higher to the former, because the scan request may not be received by the corresponding advertiser due to BLER or collisions, even though the device has been detected by the scanner. For example, for N_dev_ = 20, T_advIND_ = 176 ms, T_advInterval_ = 100 ms and a BLER of 20% all the devices are detected in less than one second and all the devices are stopped in less than two seconds. Note that in some cases an off probability is presented. This value shows the probability that all the devices become off in the T_cov_ required to detect all of them. Following the previous example, a 0.99 off means than after the first second a 100% of the devices are detected and a 99% of the devices were turned off, but it is necessary one slot more to turn off the rest.

Again, the introduction of an extra scanner improves considerably the results. Recalling also the requirements of the application, the system shall absorb peaks of less than 20 sensors per second when short advertising intervals are considered even in worse conditions (BLER 45%), with a negligible number of devices remaining active. With this configuration, the average time required to discover all devices is below 0.35 s. In addition, under good channel conditions, when short advertising intervals and frames are used, even with one scanner, we can detect up to 70 sensors in less than a second as demonstrated in the laboratory tests.

We can conclude that *interrupted scannable undirected advertisement* is clearly the best implementation alternative for all the cases, no matter the coverage area and the runner speed. On the other hand, it is important to remark that the scheme is more flexible to handle the presence under coverage of runners with different speeds. For instance, group 4 is associated to the pass through the 5 km checkpoint of a slow runner group. However, it may be possible that a runner assigned to one group actually does not belong to it. For example, a runner passing with group 4 at the 5 km checkpoint, may have started the race at the last positions and the speed of this runner is significantly higher. In this case, when *non-connectable undirected advertisement* is used, this sensor may not be detected because it passes too fast through the coverage area and there are too many interferences from the rest of the sensors inside the coverage area avoiding its detection. Nevertheless, stopping the advertisers once they are detected can solve this problem.

## 6. Conclusions

Throughout the paper we have seen that the use of BLE as a supplementary system to the current RFID solutions or even as an alternative is completely viable. One disadvantage of our proposal is that RFID systems usually employ passive sensors, whereas ours are active, although the energy consumption of the sensors is very low and they only require a minimum maintenance.

Substituting the sensors worn by the runners by Bluetooth advertisers allows to use a simple smartphone with an application as a detector. These sensors can send not only identification data, but also additional data, such as blood oxygen levels, cardiovascular monitoring, etc.

The smartphone application synchronizes all data using GPS/GLONASS/GALILEO and may employ 3G/4G or Wi-Fi networks to communicate the data to the central server. This configuration enables new services which can be offered to the users: live tracking of the race by friends and family, medical monitoring of the participants, reduction of possible cheating during the race, etc.

The proposed solutions allow for an easier deployment reducing the race costs. The system is able to detect a high number of devices, fulfilling the requirements present in real races. Additionally, we have demonstrated that with the use of a second smartphone in the same area we are able to increase the detection reliability.

## Figures and Tables

**Table 1:** Maximum coverage (m) for static and moving sensors, changing the transmission power.

Tx Power (dBm)	Static Sensors	Sensors Moving at 25 km/h
4 dBm	400 m	185 m
−8 dBm	160 m	70 m
−20 dBm	23 m	10 m
−40 dBm	6 m	2 m

**Table 2:** Sensor current consumption for different packet sizes and advertisement intervals.

Advertisement Interval	1 Data Byte	10 Data Bytes	26 Data Bytes
100 ms	17.5 µA	19.5 µA	20.25 µA
300 ms	10.5 µA	11 µA	12 µA
500 ms	9.5 µA	9.7 µA	9.9 µA

**Table 3:** *Non-connectable undirected advertisement scheme*. T_cov_ required for detecting all the devices with 100% probability vs. average required to discover all the devices, for one (N_sc_ = 1) and two (N_sc_ = 2) scanners, for BLER = 30%.

T_cov_ Required for All Detected (s)/Average Time Required to Discover All the Devices (s)												
	N_dev_		100		150		200		260		300	
T_advIND_		N_SC_	1	2	1	2	1	2	1	2	1	2
	T_advInterval_											
**176 µs**	**0.1 s**		3/1.08	2/0.70	4/1.35	3/1.04	5/1.92	4/1.41	6/2.65	5/1.92	7/3.36	6/2.35
	**0.5 s**		8/3.33	5/1.80	9/3.65	6/2.18	10/4.28	6/2.52	11/4.79	7/2.97	11/5.31	7/3.24
**376 µs**	**0.1 s**		5/1.78	3/1.23	7/2.38	6/2.15	10/4.52	6/3.40	15/7.35	11/5.62	22/10.07	16/7.68
	**0.5 s**		9/3.73	7/2.18	11/4.46	7/2.86	12/5.38	7/3.53	14/6.88	11/4.35	20/7.37	11/4.98

**Table 4:** *Interrupted scannable undirected advertisement scheme*. T_cov_ required for detecting all the devices with 100% probability vs. average required to discover all the devices, for one (N_sc_ = 1) and two (N_sc_ = 2) scanners.

T_cov_ Required for All Detected (s)/Average Time Required to Discover All the Devices (s)												
	N_dev_		100		150		200		260		300	
T_advIND_		N_SC_	1	2	1	2	1	2	1	2	1	2
	T_advInterval_											
**176 µs**	**0.1 s**		2/0.86	2/0.57	2/1.12	2/0.74	3/1.39	2/0.93	3/1.76	2/1.20	3/2.06	3/1.40
	**0.5 s**		7/3.16	6/2.26	8/3.64	6/2.62	9/3.98	7/2.90	9/4.34	7/3.13	9/4.54	7/3.30
**376 µs**	**0.1 s**		2/1.11	2/0.75	3/1.56	2/1.07	5/2.41	3/1.47	5/3.08	3/2.12	6/3.94	4/2.70
	**0.5 s**		7/3.42	6/2.45	8/3.92	7/2.85	10/4.37	7/3.15	11/4.90	7/3.56	11/5.25	8/3.80

**Table 5:** *Interrupted scannable undirected advertisement scheme*. T_cov_ required for detecting and stopping all the devices for one scanner ((x off) is the probability that all the devices becomes off in the T_cov_ required to detect all of them).

T_cov_ Required for All Detected (s)/T_cov_ Required for All Off (s)													
N_dev_		20				40				70			
T_advIND_		176 µs		376 µs		176 µs		376 µs		176 µs		376 µs	
	T_advInterval_	0.1 s	0.5 s	0.1 s	0.5 s	0.1 s	0.5 s	0.1 s	0.5 s	0.1 s	0.5 s	0.1 s	0.5 s
BLER													
**0**		1/1	5/5	1/1	5/5	1/1	5/5	1/1	6/6	1/1	5/5	2/2	5/6
**0.05**		1/1	5/5	1/1	5/5	1/1	6/6	1/2	6/6	1/2	5/6	2/2	5/6
**0.1**		1/1	5/6	1/1	5/6	1/2	6/7	1/2	6/6	2/2	6/7	2/2	6/7
**0.15**		1/2	5/7	1/2	5/6	2/2	7/7	1/2	6/8	2/2	6/8	2/2	6/8
**0.2**		1/2 0.99 off	6/9	1/2 0.96 off	6/10	2/2	7/8	2/2	6/8	2/2	7/9	2/2	7/8
**0.25**		2/2	6/9	2/2	6/10	2/2	8/11	2/2	7/11	2/3	7/10	2/3	7/11
**0.3**		2/2	7/10	2/3	7/10	2/3	8/11	2/3	7/11	2/3	7/11	2/3	7/13
**0.35**		2/2	7/12	2/3	9/13	2/3	8/11	2/3	7/14	2/4	8/13	3/4	9/14
**0.4**		2/3	8/15	2/3	9/14	2/5	8/19	2/5	7/16	2/4	9/17	3/4	9/20
**0.45**		2/4	8/22	2/5	9/18	2/4	9/20	2/5	8/18	3/5	9/17	3/6	10/20
**0.5**		2/6 0.79 off	10/25 0.89 off	2/5 0.97 off	9/21 0.67 off	3/5 0.93 off	9/22 0.54 off	3/5 0.90 off	11/25 0.75 off	3/5 0.84 off	10/23 0.48 off	3/6 0.70 off	10/26 0.41 off

**Table 6:** *Interrupted scannable undirected advertisement scheme*. T_cov_ required for detecting and stopping all the devices for two scanners. ((x off) is the probability that all the devices become off in the T_cov_ required to detect all of them).

T_cov_ Required to All Detected (s)/T_cov_ Required to All off (s)													
N_dev_		20				40				70			
T_advIND_		176 µs		376 µs		176 µs		376 µs		176 µs		376 µs	
	T_advInterval_	0.1 s	0.5 s	0.1 s	0.5 s	0.1 s	0.5 s	0.1 s	0.5 s	0.1 s	0.5 s	0.1 s	0.5 s
BLER													
**0**		1/1	4/5	1/1	5/5	1/1	5/5	1/1	5/5	1/1	6/6	1/2	5/5
**0.05**		1/1	5/5	1/1	5/5	1/1	5/5	1/1	5/5	1/1	6/6	1/2	6/6
**0.1**		1/1	5/5	1/1	5/5	1/1	5/5	1/1	5/5	1/1	6/6	1/2	6/6
**0.15**		1/1	5/5	1/1	5/6	1/1	5/5	1/1	5/6	1/1	6/6	1/2	6/6
**0.2**		1/1	5/5	1/1	5/6	1/1	5/7	1/2	5/6	1/2	6/6	1/2	6/6
**0.25**		1/2	5/6	1/2	6/6	1/2	5/7	1/2	5/6	1/2	6/7	2/2	6/7
**0.3**		1/2	5/7	1/2	6/7	1/2	5/7	1/2	5/6	1/2	6/7	2/2	6/8
**0.35**		1/2	5/7	1/2	6/7	1/2	6/8	1/2	5/7	1/2	6/9	2/2	6/8
**0.4**		1/2	5/8	1/2	6/9	1/2	6/9	1/2	6/7	1/2	7/11	2/3	6/9
**0.45**		1/2 0.91 off 0.903	5/10	1/3	6/12	1/2	6/10	2/2	7/11	2/3	7/11	2/3	7/14
**0.5**		1/3 0.76 off	6/10 0.92 off	1/3 0.69 off	6/12 0.90 off	2/3 0.99 off	6/12 0.83 off	2/3 0.99 off	7/13 0.89 off	2/3 0.97 off	7/12 0.86 off	2/4 0.95 off	7/14 0.81 off
